# 608. Systematic Fungal Biomarker Testing in Lung Transplant Recipients : Retrospective Analysis to Optimize Their Use

**DOI:** 10.1093/ofid/ofad500.674

**Published:** 2023-11-27

**Authors:** Catherine-Audrey Boutin, Michael Ison

**Affiliations:** CHUM, Université de Montréal, Montréal, Quebec, Canada; Respiratory Diseases Branch, DMID/NIAID/NIH, Derwood, MD

## Abstract

**Background:**

Serial galactomannan (GM), 1,3-β-d-glucan (BDG) and *Pneumocystis jirovecii* (PJ) testing after lung transplantation are resource consuming and have low PPV for invasive fungal infections (IFI) in asymptomatic recipients.

**Methods:**

We aim to document the added-value of mentioned reflex screenings tests in diagnosing IFI among lung transplant recipients. After IRB approval, we retrospectively collected all blood and BAL GM, BDG and PJ PCR results from patients transplanted at one large academic center from January 2015 – July 20, 2022. Manual chart review was then used to inform clinical likelihood of infection.

**Results:**

During the study period, 236 lung transplant patients were cared for by our program. Of these, 561 BAL and 56 serum GM tests were ordered. Twenty-five (4.1%) were positive ( > 1), all from BAL. Fungal cultures were requested for most BAL GM (301/343; 87.8%) and were positive for mold in 11 incidences (11/343; 3.2%). Only one was considered involved in a clinical IFI. Out of the duplicates in same BAL specimen a discrepancy in results were seen in 3.4% (7/207). 172 BDG tests were performed of which 25.6% (44/172) were positive. Among the 13 patients with serial BDG during a unique hospitalization, a mean of 2.3 tests were performed. None of the negative test repeated during the same stay became positive. Among patients with IA diagnoses and BDG testing, 5 BDG were positive (5/19; 26.3%). Three cases of IA had a positive BDG while having a negative BAL GM but had positive cultures for *Aspergillus* spp. Of the 737 BAL specimens, 577 (78.3%) had PJ testing (DFA (n=497) or PCR (n=83), or both (n=3)). None were positive. Among hospitalized patients, mean duration of stay was 31.2 d at time of testing and many had repeated testing (mean 1.8 tests). Additional results will be provided with the final analysis.

**Conclusion:**

BAL GM should be performed with fungal cultures and duplicates on same BAL probably have little additional benefit. BDG confers no added-value over BAL GM combined with cultures for the diagnosis of IA and likely can be deferred in most cases. There is limited value for systematic PJ testing on surveillance BAL of stable recipients with prophylaxis. There is especially limited diagnostic value in repeating a negative BDG or PJ testing during a stay.

**Disclosures:**

**All Authors**: No reported disclosures

